# Anti-inflammatory and anti-osteoarthritis effects of tectorigenin

**DOI:** 10.1242/bio.024562

**Published:** 2017-06-22

**Authors:** Cheng-Long Wang, De Li, Chuan-Dong Wang, Fei Xiao, Jun-Feng Zhu, Chao Shen, Bin Zuo, Yi-Min Cui, Hui Wang, Yuan Gao, Guo-Li Hu, Xiao-Ling Zhang, Xiao-Dong Chen

**Affiliations:** 1 Department of Orthopedic Surgery, Xin Hua Hospital Affiliated to Shanghai Jiao Tong University School of Medicine (SJTUSM), Shanghai 200082, China; 2 Key Laboratory of Stem Cell Biology, Institute of Health Sciences, Shanghai Jiao Tong University School of Medicine (SJTUSM) & Shanghai Institutes for Biological Sciences (SIBS), Chinese Academy of Sciences, Shanghai 200031, China

**Keywords:** Tectorigenin, Runx1, Apoptosis, NF-κB p65, Osteoarthritis

## Abstract

Osteoarthritis (OA) is a common and dynamic disease of the joints, including the articular cartilage, underlying bones and synovium. In particular, OA is considered as the degeneration of the cartilage. Tectorigenin (Tec) is known to affect many biological processes; however, its effects on articular chondrocytes remain unclear. This study aimed to assess the effects of Tec on articular cartilage. *In vitro*, Tec inhibited the expression levels of type X collagen, cyclooxigenase-2, matrix metalloproteinase (MMP)-3 and MMP-13, but enhanced the expression of Runx1, type II collagen and aggrecan in the presence of IL-1β. Meanwhile, Tec inhibited apoptosis through the Bax/Bcl-2/caspase-3 pathway, upregulating p-Bad, downregulating the Bax/Bcl-2 ratio, and activating caspase-3 compared with IL-1β treatment only. Moreover, this process was partially regulated by NF-κB P65. *In vivo*, the chondroprotective effects of Tec were assessed by establishing a model of surgically induced OA. Tec-treated joints exhibited fewer osteoarthritic changes than saline-treated joints. Meanwhile, 1.5 μg/kg Tec treatment produced a greater protective effect than 0.75 μg/kg Tec. The Osteoarthritis Research Society International (OARSI) scoring system, employed to assess histopathological grading of the models, as well immunohistochemistry for Aggrecan Neoepitope and MMP-3, further confirmed the results. In conclusion, this study showed that Tec plays a chondroprotective role in the OA process by preventing articular cartilage degeneration and chondrocyte apoptosis via the NF-κB P65 pathway.

## Abstract (precis)

**Summary:** Tectorigenin exerts anti-inflammatory and anti-osteoarthritis effects by inhibiting apoptosis in chondrocytes via the NF-κB P65 pathway.

## INTRODUCTION

Osteoarthritis (OA) is a common joint disease, regarded as a local inflammatory response caused by joint instability and accompanied by the progressive degeneration of articular cartilage (Feldmann, 2001), particularly in sites where stress exceeds the value that can be sustained by the joint. Over 12% of the aging Western population has been reported to suffer from OA, particularly those who are ≥45 years (Losina et al., 2011; Hunter and Felson, 2006). Given the aging population in the developed world, the prevalence of OA is projected to increase in the coming decades (Spector, 1993). However, the exact OA pathogenesis remains a subject of debate and research.

During the OA process, a number of inflammatory mediators have been reported (Chabane et al., 2008). Interleukin (IL) 1β is considered a main inflammatory mediator resulting in the occurrence of OA by damaging articular cartilage via NF-κB pathway activation (Chabane et al., 2008); (Goldring and Berenbaum, 1999; Newton et al., 1997). The destructive effect is also contributed by cyclooxigenase-2 (Cox-2), which produces prostaglandin E2 (PGE2), resulting in inflammation and pain in OA (Pelletier et al., 2001; Lianxu et al., 2006). Cox-2 harms the superficial layers of articular cartilage, and the NF-κB pathway also plays an important role during the process.

At present, a disease-modifying OA drug (DMOAD) that can prevent and rescue OA damage has yet to become available (Berenbaum, 2011; Kapoor et al., 2011). Common treatments for OA include nonsteroidal anti-inflammatory drugs, analgesics, locally administered corticosteroids and viscosupplementation. These medications only provide symptomatic relief to patients, who will require surgical intervention in the end. Nowadays, efforts have been made to reduce the degeneration of articular cartilage, with minimal success (Hunter, 2011).

Tectorigenin (Tec), which is an effective component of traditional Chinese medicine derived from Belamcanda chinensis, has attracted considerable interest because of its antiproliferative, anti-inflammatory and antioxidant activities (Vigne et al., 1990; Moon et al., 2006; Lee et al., 2003; Fang et al., 2008). Tec inhibits inflammatory responses caused by interferon-γ/lipopolysaccharide in murine macrophage RAW264.7 cells (Pan et al., 2008). Moreover, its anti-inflammatory effects include inhibition of NO synthase (Kimura et al., 2010) and Cox-2 expression, NO and PGE2 production, IL-1β secretion, and NF-κB signaling blockage (Pan et al., 2008; Yang et al., 2012; Kim et al., 1999).

Although Tec is involved in many biological activities, information regarding its effects on ameliorating OA remain minimal, particularly on the prevention of cartilage degeneration. Thus, our study aims to clarify the role of Tec in OA, as well as to unveil the mechanisms by which Tec affects articular cartilage degeneration. We report that Tec has anti-inflammatory and anti-osteoarthritis effects during the process of OA.

## RESULTS

### Effects of Tec on viability

**Fig. 1.**
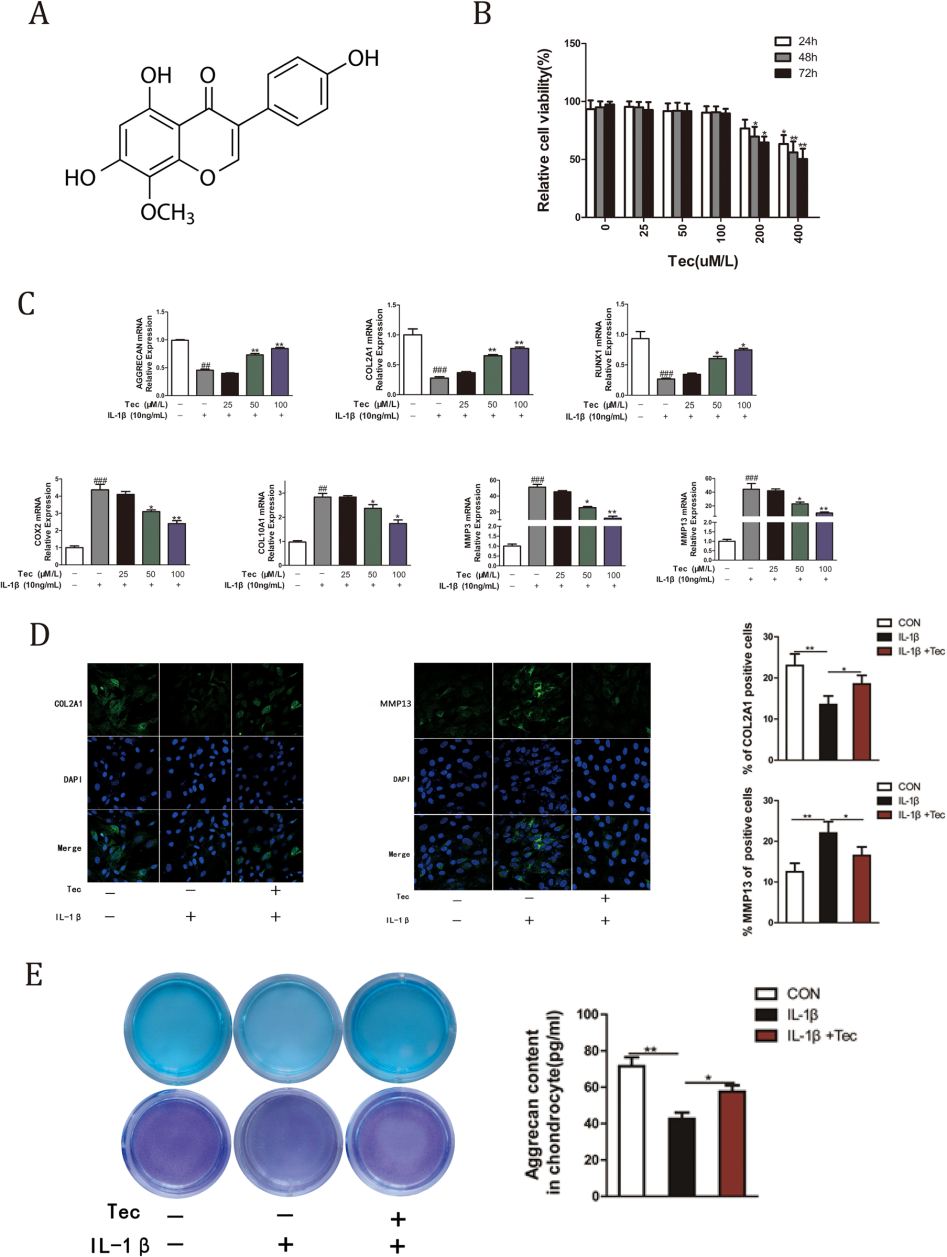
**Chondroprotective effects of Tec on chondrocytes.** (A) Chemical structure of Tec. (B) Primary cultured chondrocytes were initially plated in each well of a 96-well plate and treated with different Tec concentrations (0, 25, 50, 100, 200 and 400 μM) for 24, 48 and 72 h. Data are expressed as mean±s.d. of three independent experiments. Cells incubated in the culture medium without Tec were used as controls and considered 100% viable. (C) Primary cultured articular chondrocytes were pretreated with different Tec concentrations (25, 50 and 100 μM) for 1 h and subsequently stimulated with IL-1β (10 ng/ml) for 24 h. qRT-PCR was performed to determine the expression levels of Col2a1, Col10a1, MMP-3, MMP-13, Runx1, Cox-2 and Acan in chondrocytes. Data are expressed as mean± s.d. *P<0.05, **P<0.01, compared with cells stimulated with IL-1β alone. ^##^*P*<0.01, ^###^*P*<0.001, compared with cells cultured without Tec and IL-1β. (D) Primary cultured articular chondrocytes were treated with or without 10 ng/ml IL-1β and 100 μM Tec for 24 h. Expression levels of Col2a1 and MMP-13 were detected via immunofluorescence staining. The bar graphs show the percentages of Col2a1- and MMP-13-positive cells. (E) Alcian Blue and Toluidine Blue staining in chondrocytes at 14 days cultured with or without IL-1β (10 ng/ml) and Tec (100 μM) for 72 h. Alcian Blue and Toluidine Blue staining was quantified to determine chondrocytes’ aggrecan content.

The chemical structure of Tec is shown in Fig. 1A. The effects of different concentrations (0, 25, 50, 100, 200 and 400 μM) on primary cultured chondrocytes were assessed via 3-(4,5-dimethylthiazol-2-yl)-2,5-diphenyltetrazolium bromide (MTT) assay. Tec concentrations ≥200 μM exhibited mildly toxic effects on chondrocytes, particularly at 48 h and 72 h. The effect heightened at 400 μM after 48 h (Fig. 1B, *P*<0.05). Thus, 25, 50 and 100 μM Tec concentrations were used for the *in vitro* tests.

### Chondroprotective effects of Tec on primary cultured articular chondrocytes

To determine the effects of Tec on primary cultured articular chondrocytes, the chondrocytes were pre-incubated with Tec for 1 h, then stimulated with IL-1β for 24 h. IL-1β-stimulated chondrocytes exhibited upregulation of Col10a1, Cox-2, MMP-3 and MMP-13 levels, but downregulation of Runx1, Col2a1 and Acan expression levels (Fig. 1C, ##*P*<0.01, ###*P*<0.001). Tec inhibited the IL-1β-mediated induction of Col10a1, Cox-2, MMP-3 and MMP-13 levels, and enhanced levels of Runx1, Col2a1, and Acan with IL-1β (Fig. 1C, *P<0.05, **P<0.01). Additionally, these changes were more significant at 50 μM and 100 μM concentrations than at 25 μM. Immunofluorescence staining was conducted to further demonstrate the effects of Tec on Col2a1 and MMP-13 expression (Fig. 1D). IL-1β treatment dramatically decreased Col2a1 expression and increased MMP-13 expression. Nevertheless, Tec reversed the trend at 100 μM. Furthermore, 100 μM Tec further enhanced cartilage matrix synthesis (as indicated by Alcian Blue and Toluidine Blue staining) compared with IL-1β treatment only (Fig. 1E).

### Tec downregulated Cox-2 and upregulated Runx1 via NF-κB P65

**Fig. 2.**
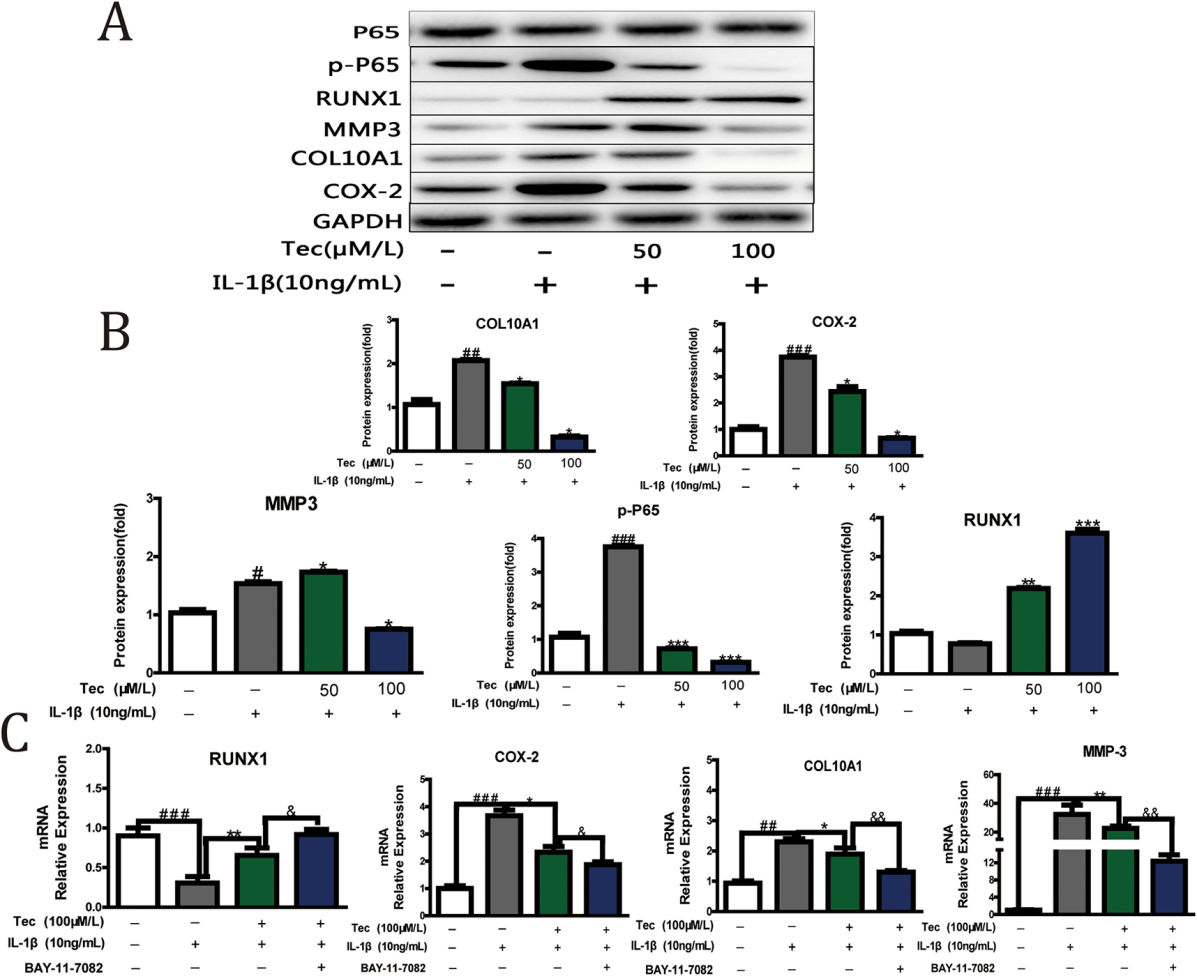
**Tec downregulated Cox-2 and upregulated Runx1 via NF-κB P65.** (A) The primary cultured articular chondrocytes were treated with or without IL-1β (10 ng/ml) and Tec (50 μM and 100 μM) for 72 h, and expression levels of Col10a1, MMP3, Cox-2, Runx1 and p-P65 were analyzed via western blotting. GAPDH was used as a loading control. (B) Densitometric analysis of the immunoblot band intensities for Col10a1, MMP-3, Cox-2, Runx1 and p-P65, normalized to GAPDH. (C) qRT-PCR was performed to determine the expression levels of Col10a1, MMP-3, Runx1 and Cox-2 by using a specific NF-κB inhibitor, BAY-11-7082. Data are expressed as mean±s.d. of three independent experiments. *P<0.05, **P<0.01, ***P<0.001, compared with cells stimulated with IL-1β only; ^#^*P*<0.05, ^##^*P*<0.01, ^###^*P*<0.001, compared with cells cultured without Tec and IL-1β; ^&^*P*<0.05, ^&&^*P*<0.01, compared with cells stimulated with Tec and IL-1β; *n*=3.

To evaluate which pathway was involved in the chondroprotective effects of Tec, we conducted an experiment on Tec (50 μM and 100 μM) at the protein level. Western blot and densitometric analyses showed that Tec downregulated the expression levels of P-p65, MMP-3, Col10a1 and Cox-2, but upregulated Runx1, particularly at 100 μM, when compared with IL-1β treatment only. The results indicated that NF-κB P65 was involved in the mechanism underlying the chondroprotective effects of Tec (Fig. 2A,B). By using a specific NF-κB inhibitor, BAY-11-7082, a more obvious trend of Col10a1, MMP-3, Runx1 and Cox-2 expression was detected compared with those of the IL-1β and Tec treatment group. These results further demonstrated that Tec exerted chondroprotective effects partially through NF-κB P65 (Fig. 2C).

### Tec exerts inhibitory effects on IL-1β-induced apoptosis in chondrocytes through the NF-κB pathway

**Fig. 3.**
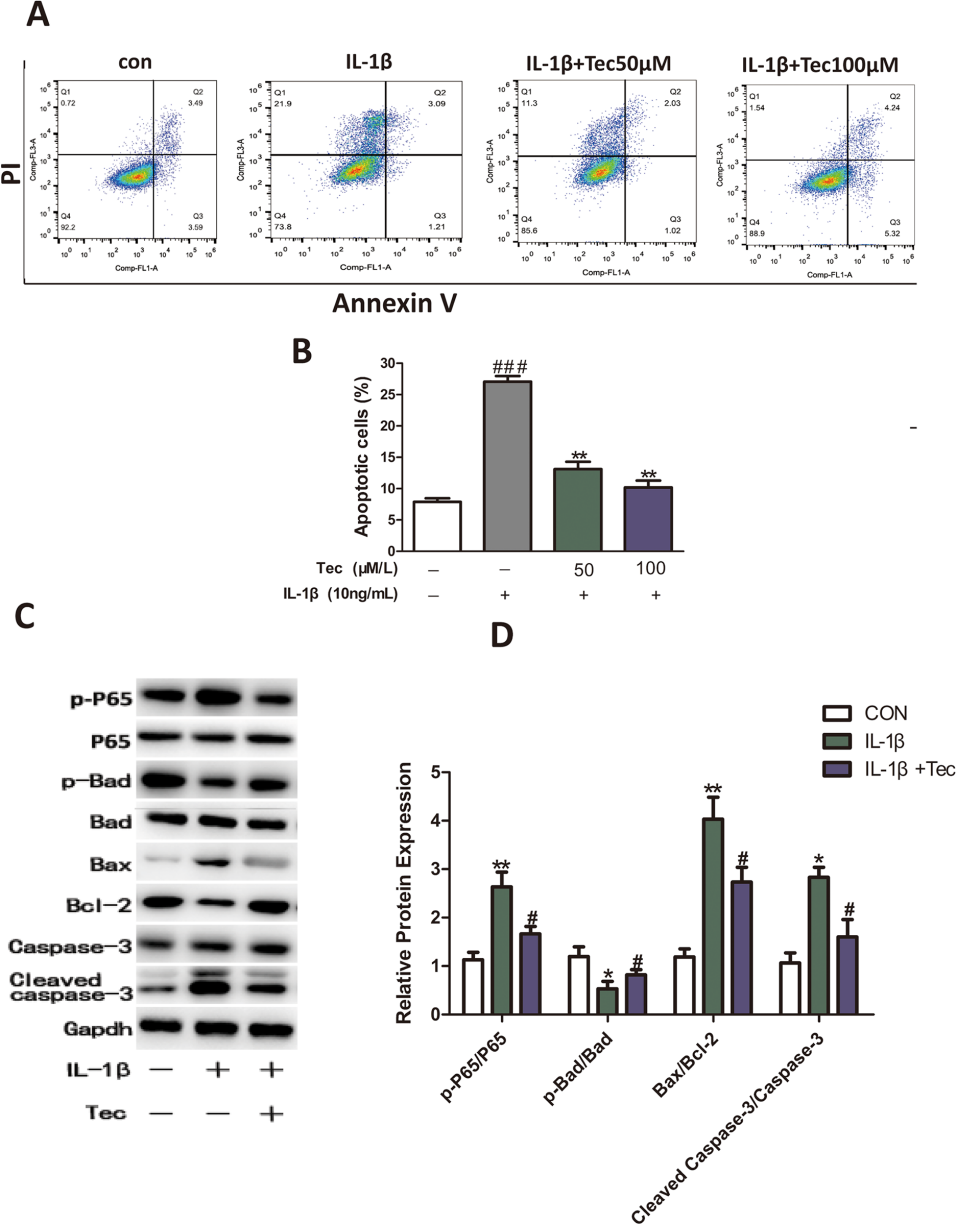
**Effects of Tec on IL-1β-induced apoptosis in chondrocytes.** (A) Apoptosis assayed via flow cytometry using annexin V/PI double staining. (B) Quantitative analysis of apoptotic chondrocytes in each group. (C) Apoptosis-related proteins were analyzed through western blotting after Tec treatment (50 μM) and without Tec treatment. (D) Quantitative analysis of expression of p-Bad, Bax, Bcl-2 and caspase-3. Data are expressed as mean±s.d. of three independent experiments. Data of the treatment group are expressed as fold change versus that of control group (labeled as ‘1.00’) after normalizing to GADPH. *P<0.05, **P<0.01, ***P<0.001, compared with cells cultured without Tec and IL-1β; ^#^*P*<0.05, ^##^*P*<0.01, ^###^*P*<0.001, compared with cells stimulated with IL-1β only.

To elucidate the effects of Tec on apoptosis in chondrocytes, we performed an experiment on chondrocytes that were pre-incubated with Tec for 1 h, then stimulated with IL-1β for 24 h, after which flow cytometry was conducted and the proteins examined. Based on the quantitative results, we observed less apoptosis in chondrocytes (Fig. 3A,B) and decreased p-p65, Bax and caspase-3 protein levels, and increased Bcl-2 and p-Bad protein levels, in the Tec group compared with the IL-1β treatment only group (*P*<0.05) (Fig. 3C,D). This result showed that Tec played a protective role in IL-1β-induced apoptosis in chondrocytes through the NF-κB pathway

### Protective effects of Tec on surgically induced OA

**Fig. 4.**
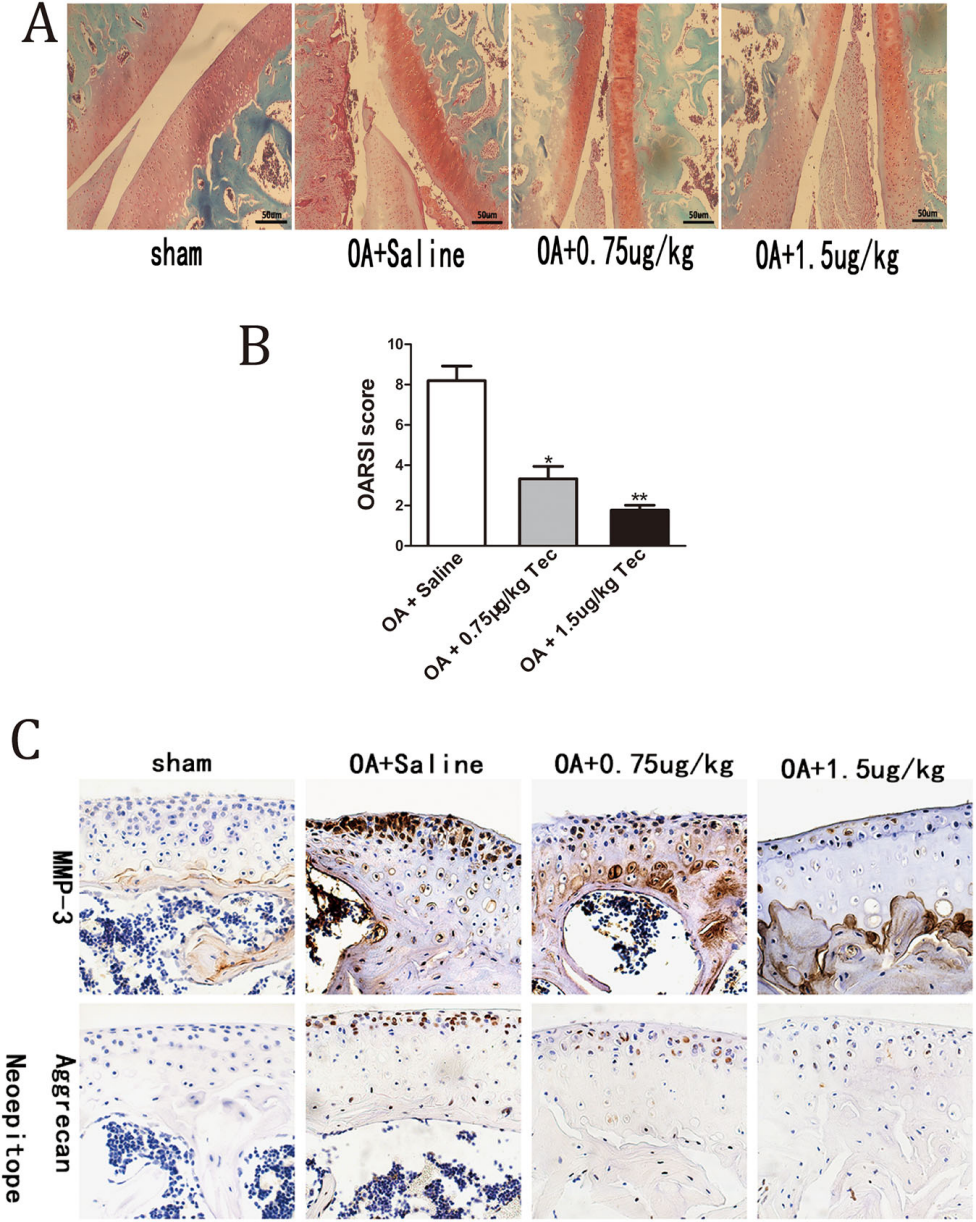
**Effects of Tec treatment on surgically induced OA.** (A) Knee joints were harvested at 6 weeks postsurgery and analyzed histologically via Safranin O−fast green staining. Representative images are shown. (B) OA of the femoral and tibial cartilage was scored histologically using the OARSI scoring system at 8 weeks postsurgery. (C) Immunohistochemical staining of Aggrecan Neoepitope and MMP-3. Data are expressed as mean±s.e.m. (*n*=10). *P<0.05, **P<0.01, compared to treatment with saline. Scale bars: 50 μm.

We also assessed the effects of Tec in an OA mouse model to test if Tec exerts protective effects *in vivo*. Periodic Tec or saline was injected into the knee joints (after OA model establishment) for 6 weeks. The representative histological sections showed that the Tec-treated joints had less osteoarthritic damage than the saline-treated group. Furthermore, the protective effects were more evident in the 1.5 μg/kg mice group than in the 0.75 μg/kg mice group. Histopathological grading using the Osteoarthritis Research Society International (OARSI) scoring system (Glasson et al., 2010) further confirmed the result (Fig. 4A,B). Immunohistochemistry for Aggrecan Neoepitope and MMP-3 indicated decreased Aggrecan Neoepitope and MMP-3 in the Tec group (0.75 μg/kg) compared with the saline-treated group. Moreover, the trend of decreased Aggrecan Neoepitope and MMP-3 was more significant in the Tec group (1.5 μg/kg) (Fig. 4C).

## DISCUSSION

Tec, which inhibits the inflammation of acute lung injury in mice, is derived from the Chinese herb Belamcanda chinensis (Ma et al., 2014). Another study has shown Tec antibacterial activity against methicillin-resistant *Staphylococcus aureus* (Joung et al., 2014). Therefore, Tec may represent a potentially effective option to treat inflammation. However, the mechanisms of Tec in OA have yet to be determined. The current research aimed to investigate whether Tec has anti-inflammatory activity against OA. Our data showed that Tec suppressed the OA process. In particular, Tec prevented chondrocyte degeneration and chondrocyte apoptosis without inducing cartilaginous hypertrophy.

OA is a common disease and its incidence increases with age. At present, OA is managed through various treatment modalities, including pharmacological and nonpharmacological therapies (McAlindon et al., 2014; Hochberg et al., 2012). Until now, however, no effective treatment has been found. Most therapeutic methods, particularly the DMOAD, have attempted to prevent or delay articular cartilage degeneration and achieved minimal success (Hunter, 2011). Thus, repairing damaged articular cartilage may provide a another way to treat OA patients. In the present study, we observed that Tec is a new potential candidate for OA treatment.

Previous reports showed that OA development may result from hypertrophic differentiation of articular chondrocytes (Hunter and Felson, 2006). This result is further supported by our findings that Tec may improve OA without inducing hypertrophy, as determined by the assessment of increased expression of Runx1, which induces chondrogenic differentiation and suppresses subsequent hypertrophy (Kimura et al., 2010; Wang et al., 2005), as well as decreased hypertrophic marker expression (Collagen type X) (Fig. 3A,B). In OA, however, articular cartilage abnormally suffers from hypertrophy and is followed by endochondral ossification, resulting in arthrosis degeneration (Lories and Luyten, 2011). Multiple signaling pathways, including WNT and NF-κB, are involved in the hypertrophic differentiation of articular chondrocytes, (Miller, 2002; Bradley and Drissi, 2010). The outcomes were consistent with our finding that Tec partially inhibited NF-κB p65, accompanied by increasing Runx1 levels and declining Collagen X levels. Moreover, NF-κB inhibitor BAY-11-7082 intensified this trend (Fig. 2C).

Chondrocytes, the only cells in articular cartilage, maintain the dynamic balance between synthesis and degradation of the extracellular matrix (ECM). Apoptotic cell death has been observed in OA cartilage, related to matrix degradation and calcification, suggesting a role in OA pathogenesis. A previous study has shown that Caspase-3 is regarded as an main factor in the process of occurrence of apoptosis (Yu et al., 2015). Furthermore, the Bcl family, which can be divided into anti-apoptotic proteins (Bcl-2, Bcl-xL, Bcl-w and Mcl-1) and pro-apoptotic proteins (Bax, Bad, Bak, Bik and Bid), have been reported to play an important role during apoptosis (Koff et al., 2015). In our study, we observed that Tec inhibited apoptosis via the Bax/Bcl-2/caspase-3 pathway, upregulating p-Bad, downregulating Bax/Bcl-2 ratio and activating caspase-3, when compared to IL-1β treatment only. Of particular interest, Tec decreased p-p65 protein levels, compared to IL-1β treatment only (Fig. 3C). The result showed that Tec plays a protective role in IL-1β-induced apoptosis in chondrocytes partially through the NF-κB pathway. However, inhibitors of apoptosis can have potential side effects, such as carcinogenesis (Hwang and Kim, 2015). Thus, a means of modifying apoptosis safely to inhibit or suppress apoptosis in OA remains obscure.

In conclusion, this study demonstrated the positive role of Tec in OA restriction. *In vitro*, we identified Tec as an apoptosis inhibitor in chondrocytes, partially via the NF-κB P65 pathway. *In vivo* investigation showed that Tec could ameliorate manifestation of knee joint cartilage in a model of OA. The outcomes indicate that Tec may be a potential therapeutic candidate for OA treatment.

## MATERIALS AND METHODS

### Reagents

Tec (purity, >98%; CAS number, 548-77-6; molecular weight, 300.26) was supplied by Chengdu Must Bio-Technology (Chengdu, China).

### Experimental animals

Animal experiments were conducted according to protocols approved by the Laboratory Animal Welfare and Ethics Committee of Shanghai Jiaotong University (Shanghai, China).

### Primary cell culture

The primary chondrocytes were isolated from the femoral condyles and tibial plateau of male Sprague Dawley (SD) rats (160–180 g). Rat articular cartilage was cut into small fragments and digested first with 0.25% trypsin (Gibco Invitrogen, Carlsbad, CA, USA) for 30 min at 37°C and then with 0.2% collagenase (Sigma-Aldrich) for 5 h at 37°C. After dissociation, the cell suspension was filtered through a 40 μm cell strainer (BD Falcon, Bedford, MA, USA), and the cells were collected via centrifugation at 800× ***g*** for 10 min. Chondrocytes were resuspended in DMEM/F-12 medium (Gibco Invitrogen) supplemented with 10% fetal bovine serum (Gibco Invitrogen). The primary chondrocytes were cultured following a previously presented method (Gosset et al., 2008). The chondrocytes were seeded in six-well plates (2×10^5^/well), and the sub-confluent cells were pre-incubated with five Tec concentrations (25, 50, 100, 200 and 400 μM, as determined in the preliminary tests) for 1 h, then stimulated with IL-1β (10 ng/ml) for 24 h. The cells were harvested, and the related gene mRNA expression and protein levels were determined to assess Tec concentrations, which were used in subsequent experiments.

### MTT assay

To determine the cytotoxicity of Tec on chondrocytes, 3-(4,5-dimethylthiazol-2-yl)-2,5-diphenyltetrazolium bromide (MTT) (#M5655, Sigma-Aldrich) assay was applied based on a previously described protocol (Ma et al., 2014).

### Apoptosis assay

Annexin V and Propidium Iodide (PI) double staining was conducted to determine the apoptosis level caused by the Tec. Chondrocytes were seeded at 2×10^5^ cells/well in six-well plates and incubated for 24 h. The images were recorded using a fluorescent inverted microscope. The remainder of the cells were collected and resuspended in 1× binding buffer at ∼1×10^6^ cells/ml. The cell suspension was added to 5 μl annexin V and 1 μl PI (#V23200, Life Technologies, Carlsbad, CA, USA), and incubated for 15 min in the dark at room temperature. Then, 400 μl 1× binding buffer was added to the suspension, and the samples were examined with a flow cytometer (FACSA, BD Biosciences) at a wavelength of 488 nm.

### Surgical induction of OA

Animal handling and experimental procedures were performed following approval from the Institute of Health Sciences Institutional Animal Care and Use Committee. Eight-week-old male SD rats (200 g) were randomly divided into four groups: (1) 1.5 μg/kg Tec-treated animals, (2) 0.75 μg/kg Tec-treated animals, (3) saline-treated animals, and (4) sham group (*n*=10 mice in each group). OA was induced via medial collateral ligament transection and medial meniscal tear on the knee joints, as previously described (Moore et al., 2005). The animals were anesthetized, and surgery was performed to transect the medial collateral ligament and cut through the full thickness of the medial meniscus to induce joint destabilization of the right knee. Sham animals underwent the same surgical procedure without any ligament transection or meniscal tear. After surgery, each rat was given penicillin once a day for the first 3 days. For intra-articular injection, Tec was dissolved in 100 μM in DMSO (#94563, Sigma-Aldrich) before being used in sterile saline (0.9% NaCl, JinTong Pharmaceutical Factory, Shanghai, China). The mice were re-anesthetized and administered with a 10 µl intra-articular injection of Tec or saline immediately after surgery every 5 days for 8 weeks. The animals were sacrificed at 8 weeks post-surgery, and samples of the knee joints were collected for further molecular and histological analyses.

### Real-time PCR and western blot analysis

Quantitative real-time polymerase chain reaction (qRT-PCR) and western blot analysis was based on a previously described protocol (Wang et al., 2017). The primers were produced by Sangon Biotech (Shanghai, China). Primer sequences are listed in Table S1.

### Statistical analysis

All numerical data are expressed as mean±s.d. Statistical differences among groups were analyzed by one-way ANOVA. All statistical analyses were performed with SPSS software (SPSS Inc., Chicago, USA), version 16.0. Statistical differences between two groups were determined by the Student's *t*-test; *P*<0.05 was considered statistically significant.
